# Do age and birth cohorts influence trends in the job demand–control model, self-rated health, and perceived stress in Sweden?

**DOI:** 10.1177/14034948251381537

**Published:** 2026-02-16

**Authors:** Paraskevi Peristera, Anna Andreasson, Göran Kecklund, Mats Lekander, Hugo Westerlund

**Affiliations:** 1 Division of Psychobiology and Epidemiology, Department of Psychology, Stockholm University, Sweden; 2 Division of Psychology and Osher Centre for Integrative Health, Department of Clinical Neuroscience, Karolinska Institutet, Stockholm, Sweden

**Keywords:** Psychosocial work environment, perceived long-lasting stress, sustained emotional stress, self-rated health, age–cohort effects, hierarchical APC-growth curve models

## Abstract

**Objectives:**

People in Sweden have internationally good health, work environment and quality of life, a picture that has lately been questioned because of the sharp increase in stress-related diagnoses, especially among young women. This study aims to better understand how stress and psychosocial work stressors vary across age and birth cohorts using data collected between 2008 and 2018.

**Methods:**

Using hierarchical age-period-cohort-growth curve models and data from the Swedish Longitudinal Occupational Survey of Health, we estimated the age trajectories of job demands, job control, perceived long-lasting stress, sustained emotional stress and self-rated health by birth cohort. Separate analyses by sex and occupational status were also conducted.

**Results:**

Job demands, perceived long-lasting stress and sustained emotional stress decreased with age, while self-rated health deteriorated. Statistically significant cohort and age × cohort interactions suggest that more recent birth cohorts have both a) higher job demands, lower job control, more sustained emotional stress and worse self-rated health, and b) a less favourable development with age for all outcomes. However, these cohort differences are primarily driven by younger cohorts, and they are less pronounced or inconsistent among older cohorts. Occupational status and gender seem to modify some of these developments.

**Conclusions:**

**This study provides some support for inter-cohort changes in the work environment and health, predominantly among younger cohorts. More recent cohorts generally report poorer health and work environment along with less favourable development with age. Nevertheless, these patterns are not consistent across all age group comparisons and robust cohort comparisons are primarily possible among adjacent cohorts. It is, however, unlikely that these changes can be attributed solely to a general deterioration in working life affecting all workers equally, as the phenomenon appears largely confined to younger workers. Future studies should explore how the combined demands from several spheres of life may better explain these age and cohort differences.**

## Key messages

1. This study examines age trajectories of job demands, job control, perceived long-lasting stress, sustained emotional stress and self-rated health by birth cohort over a 10-year period in Sweden.
2. The most recent cohorts report poorer health and work environment along with less favourable development with age. Occupational status and gender appear to modify some of these patterns. However, these findings are primarily applicable to younger cohorts and broader generalizations should be made with caution.
3. Further studies should disentangle between age and cohort effects in order to investigate how demands from several spheres of life might explain these differences.

## Introduction

Psychosocial risks and work-related stress are among the most challenging issues in occupational safety and health. Despite a long tradition of workplace health promotion in Sweden, there is a growing concern about the development of the psychosocial work environment owing to the rise in sickness absences with stress-related mental diagnosis over the last decade [1,2]. There is also an increased awareness of the issue owing to the introduction of new provisions on the organizational and social work environment by the Swedish Work Environment Authority (AFS2015:4), which were largely based on a widely publicized report on increasing work-related complaints [3]. It remains, however, an open question to what extent the increase in stress-related complaints reflects an actual deterioration of the psychosocial work environment, in terms of job demands and job control. At least part of the increase in sickness absence due to stress-related diagnoses can be explained by changes in the regulations for determining sickness benefits in Sweden, and the increasing use of psychiatric diagnoses instead of musculoskeletal ones [1].

In fact, there are methodological challenges in appraising the development of the psychosocial work environment, perceived stress and self-rated health in a population. The main challenge, in examining longitudinal trends, is to disentangle age and birth cohort effects [4]. Birth cohort effects refer to differences in the experiences and outcomes of individuals born and growing up in different historical time periods. There may be effects that are unique to a particular birth cohort or that accumulate over a lifetime. For example, cohorts might differ in terms of basic health, ethnic background, educational level, job expectations, types of occupation, financial resources, coping style and political outlook, all of which might affect how they rate their work environment, stress and health. In addition to cohort effects, there are age effects and the consequences of growing older, independent of birth cohort and calendar time, which are difficult to disentangle from cohort and period effects. Repeated cross-sectional studies cannot determine whether changes in job demands or self-rated health and stress are due to cohort effects, because comparing two cohorts at the same point in time means that one is older than the other. In order to be able to compare different cohorts at the same chronological age, longitudinal data spanning the age difference between cohorts are required. As a result, most previous studies on the development of the psychosocial work environment and stress do not take age and cohort effects into account because of a lack of data to support this type of analysis.

Previous research has shown inconsistent results regarding the development of the psychosocial work environment over time. Some studies have found that Swedish employees experienced significant increases in job demands and decreases in job control [5–8] while other studies have found stable or positive changes in job demands and resources [9,10]. Previous studies have also found differences between occupations and between men and women. For example, one recent study found that care workers experienced higher job demands and lower job control than other occupational groups [11] while another study found that job demands decreased more steeply with age for white-collar workers than for blue-collar workers [12]. In addition, previous studies suggest that women experience higher job demands than men [13–15].

There is also mixed evidence for an increase in stress-related mental health problems (e.g. anxiety, depressive symptoms, exhaustion). Some earlier studies have shown increases in self-reported depressive symptoms, anxiety and psychiatric service use over time, particularly among young women [16–26]. However, other studies have reported stable levels of clinically significant depression and anxiety [22,23]. With regard to stress levels, studies describing the development of perceived long-lasting stress at a total population level are rare. The few existing studies and some recent reports suggest increased stress levels over time [15,27–30]. For instance, one Swedish study found that perception of high mental stress increased between 1980 and 2016, for both 38- and 50-year-old women [27] while another study found that women in Germany have higher levels of stress compared with men [28].

Self-rated health is another health-related outcome that should be considered in relation to poor psychosocial working conditions [26,31]. A previous paper examining longitudinal trends in self-rated health in Sweden suggests that self-rated health declines with increasing age [32]. Another study, focusing on young adults aged between 25 and 34 years old, in northern Sweden, found an increase in the proportion of women rating their health as poor in more recent years whereas in men the proportion rating good increased [33]. Some earlier studies have also examined self-rated health across birth cohorts but the evidence is still mixed and mostly from outside Sweden [34–36]. To our knowledge, there is only one study in the Swedish context that has examined the trends in self-rated health by age and cohort, and it suggests that self-rated health has improved for the elderly but deteriorated for the youngest [37]. To date, we are not aware of any studies that have used an age–cohort analysis to examine the trajectories of work stress according to the demand–control model, perceived long-lasting stress and sustained emotional stress. In summary, previous research has incompletely explored the development of the psychosocial work environment, perceived stress and self-rated health over time because it has not examined age and cohort differences. Assessing age and cohort effects is important because it allows us to distinguish changes over time within individuals (age effects) from differences between individuals at baseline (cohort effects).

The aim of this study is to better understand the development of stress and psychosocial work stressors by using a hierarchical age-period-cohort (APC)-growth curve modelling approach. This approach is used to estimate birth cohort effects in the age trajectories of job demands, job control, perceived long-lasting stress, sustained emotional stress and self-rated health over a 10-year period. Multilevel growth curve analysis has been widely used to assess age-dependency analysis in standard single-cohort longitudinal panels [38]. The APC-growth curve modelling approach extends multilevel growth curve analysis when analysing multiple cohort panel data [4]. The hierarchical APC-growth curve models (HAPC-GCMs) aim to overcome the problem of simultaneously estimating age, period and cohort effects by explicitly incorporating cohort effects and implicitly incorporating period effects in the form of age-by-cohort interaction. A second objective is to examine whether trends differ by gender and occupational status.

## Methods

### Study population

The data were derived from the Swedish Longitudinal Occupational Survey of Health (SLOSH), an approximately nationally representative cohort of the Swedish working population. SLOSH was launched in 2006 [39] and has been conducted biennially until 2022 and annually thereafter. It consists of respondents from the Swedish Work Environment Surveys (2003–2019), who in turn are sampled from the Labour Force Survey by Statistics Sweden. Prior to the 2022 wave, SLOSH consisted of two questionnaires, one for people in paid work for ⩾30% of full-time and another for people not in paid work. SLOSH was approved by the Regional Research Ethics Board in Stockholm with additional approval from the Swedish Ethical Review Authority.

The sample used in this study is based on SLOSH participants who completed the work questionnaire at least three times between 2008 and 2018 (*N*=17,774). We first included participants from the 2008 wave as this was the first time that the stress questions were available in SLOSH. We did not include waves later than 2018, in order to avoid potential impact of the Covid-19 pandemic and the transition to an online-first format. Because of the interest in job-related demands, the analyses were restricted to individuals aged 25 to 65 years (*n*= 17,730).

### Variables

*Job demands and control* were measured using the Swedish Demand–Control–Support Questionnaire [40–42], in which items were scored on a Likert scale, from 1= ‘yes, often’ to 4 = ‘no, never or basically never’, with four items assessing job demands (work very fast, too much effort, enough time (reverse-coded), conflicting demands) and five items assessing job control (control over what to do at work, control over how to do the work, learn new skills, high level of skill or expertise, repetitive work (reverse-coded)). Job demands and job control were calculated as the mean values of the above items with higher values indicating higher job demands and higher job control.

*Perceived stress* was assessed using a scale developed for the SLOSH study, which asked whether, in the last three months, the respondent: a) had days feeling aroused all the time; b) had days feeling very pressed (and the limit they could tolerate), c) had difficulties relaxing during free time; d) had been often tensed; e) had worrying thoughts; f) had been often restless; g) had not felt rested after some days of relaxation; h) had days feeling stressed all the time. The response alternatives were ‘not at all’ scored as (1), ‘sometimes’ (2), ‘quite often’ (3) and ‘almost always’ (4). Exploratory factor analysis carried out on the baseline data was applied in order to appropriately select the questions that could be combined to create an index of stress (results available on request). Based on the results of the factor analysis, two factors were identified. The first factor included items a, b and h and was labelled *perceived long-lasting stress*. Åkerstedt et al.. (2015) measured perceived stress in the same way as a complement to the traditional work demand scale [43]. The second factor included the remaining five items (c–g) and was labelled sustained emotional stress. Perceived long-lasting stress and *sustained emotional stress* were calculated as the mean of the previously described items, with higher values indicating more perceived long-lasting stress and sustained emotional stress, respectively.

Self-rated health was measured with a single item: ‘How would you rate your general state of health?’ answered on a five-point scale with 1 = ‘very good’, 2 = ‘quite good’, 3 = ‘neither good nor bad’, 4 = ‘quite poor’ and 5 = ‘very poor’. The validity and reliability of this question has been demonstrated in several studies and the item is considered a reliable and valid global health measure [44,45]. To facilitate interpretation of the results, we used a reversed scale in the analyses with higher values indicating better health in the analyses.

Age in years was derived from administrative registers at the time of the survey interview. The period was represented by the SLOSH waves from 2008 to 2018. Birth year was obtained by subtracting age from the survey year and ranged from 1943 to 1993. Birth cohort was categorized into 10-year periods from 1943 to 1993 for convenience: 1 = 1943–1952; 2 = 1953–1962; 3 = 1963–1972; 4 = 1973–1982; 5 = 1983–1993. The distribution of participants by cohort group was 17.77% (*n* = 3055) for birth group 1; 35.98% (*n* = 6187) for birth group 2; 29.59% (*n*=5087) for birth group 3; 15.85% (*n* = 2726) for birth group 4; 0.81% (*n* = 675) for birth group 5. Sex and occupational status were derived from linkage to registry data. Sex was binominal-coded into men (0) and women (1). Occupational status was also binomial-coded into blue-collar workers (0) and white-collar workers (1), where white-collar workers included the occupations 1 = ‘Legislators, senior officials, managers’, 2 = ‘Professionals’, 3 = ‘Technicians’; blue-collar workers included the occupations 4 = ‘Clerks’, 5 = ‘Service workers’, 6 = ‘Agricultural and fishery workers’, 7 = ‘Craft workers’, 8 = ‘Machine Operators’, 9 = ‘Elementary occupations’; following the Statistics Sweden official classification of occupations.

### Statistical analyses

All analyses were carried out in Stata 16.1. First, summary statistics are presented for the entire sample. Second, trajectories of a) job demands and job control, and b) perceived long-lasting stress, sustained emotional stress and self-rated health were estimated through HAPC-GCMs of inter-cohort age change [4], for the full sample as well as stratified by gender and occupational status (white-collar *vs* blue-collar workers). To assess the potential impact of socio-structural composition, we additionally estimated adjusted models for the full sample including gender, occupational type, educational level, and country of birth as covariates. The APC-growth curve models are growth curve models, applied to multi-cohort panel data and are therefore used differently from classical growth models, which examine change in a single cohort over time [4,46]. This approach has the advantage of allowing data that are unbalanced in time, meaning all individuals can contribute to the estimation of trajectories regardless of the number of waves, as each observation is included in the person-year dataset [38]. In this approach missing values are treated using maximum likelihood estimation.

The HAPC-GCMs used in this study have a two-level structure with level 1 units being the repeated observations over time, which are nested within individuals who are level 2 units (see Appendix 2). For each of the outcomes, we examined the effects of age and cohort and also tested for a statistical interaction to assess whether the age trajectory differed by cohort. Models with either linear or quadratic age effects were tested in order to determine whether a simple, constant relationship between age and the outcomes was sufficient or whether a more complex relationship was needed. The models with the best fit were selected as the most appropriate. Models with quadratic age terms were selected if the inclusion of these quadratic terms significantly improved the model fit, regardless of their statistical significance. Cohort effects were linear for all outcomes.

**Figure 1.**
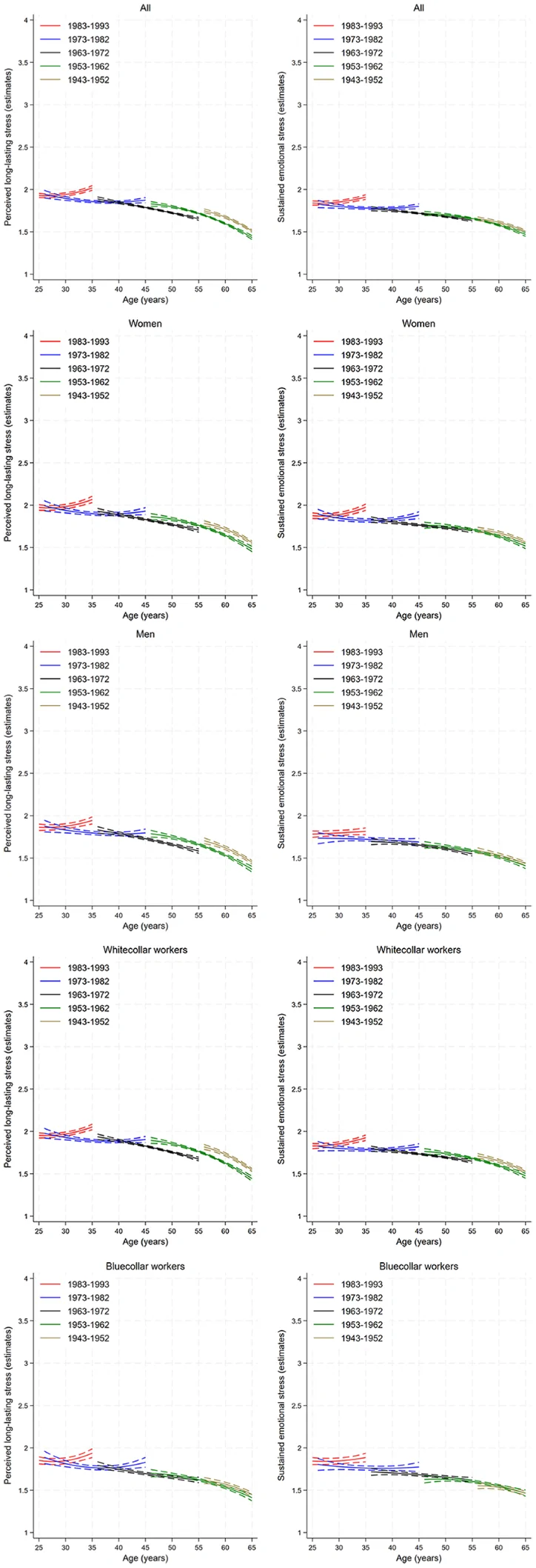
Predicted age trajectories and 95% confidence intervals of perceived long-lasting stress, sustained emotional stress (range 1–4) from the unadjusted models. The panels show trajectories for the whole sample as well as stratified by sex and occupational status: The *x*-axis represents age (in 10-year intervals). The *y*-axis represents the estimated value of the outcome variable. The cohorts are illustrated as follows: brown line for the 1943–1952 cohort, green line for the 1953–1962 cohort, black line for the 1963–1972 cohort, blue line for the 1973–1982 cohort and red line for the 1983–1993 cohort. Data source: The Swedish Longitudinal Occupational Survey of Health, 2006–2018, total number of individuals, *N*=17,730.

**Figure 2.**
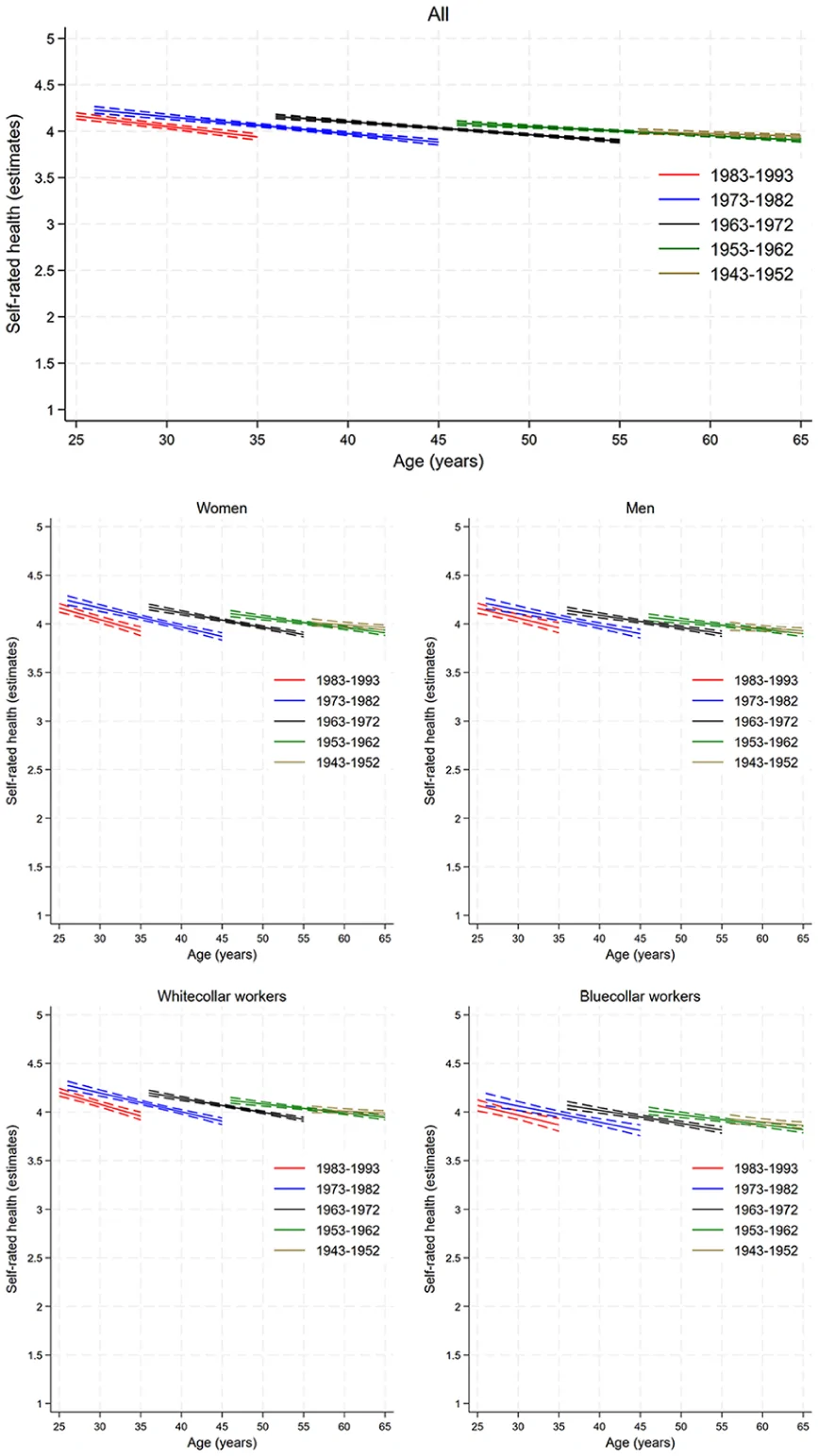
Predicted age trajectories of self-rated health (range 1–5) and 95% confidence intervals from the unadjusted models. The panels show trajectories for the whole sample as well as stratified by sex and occupational status: the *x*-axis represents age (in 10-year intervals). The *y*-axis represents the estimated value of the outcome variable. The cohorts are illustrated as follows: brown line for the 1943–1952 cohort, green line for the 1953–1962 cohort, black line for the 1963–1972 cohort, blue line for the 1973–1982 cohort and red line for the 1983–1993 cohort. Data source: The Swedish Longitudinal Occupational Survey of Health, 2006–2018, total number of individuals, *N*=17,730.

Age was centred around the median age of the 10-year cohort to which the individual belonged (cohort-median centred). This centring allows the intercept to be interpreted as the estimated value of the outcome variable at the median age within each cohort. This process involves subtracting the cohort median from the individual ages, resulting in both negative and positive values for the centred age variable. However, this centring is recommended as it protects the estimate from bias associated with systematic variation in mean age between cohorts [47]. Cohorts were defined by a 10-year age range that accounted for the variation associated with age and period within this 10-year range. The adjusted estimates are shown alongside the unadjusted estimates in Table II. To illustrate the results graphically, we plotted the unadjusted predicted age trajectories of the outcomes for each cohort (Figures 1 and 2) as well as the observed age trajectories (Figures 4 and 5 in Appendix 1). In Appendix 1, Tables III and IV show the estimated parameters from the unadjusted fitted growth models when stratified by gender and occupational type and Tables V and VI show the values of the random effects-variance components and measures of goodness-of-fit for the unadjusted models.

## Results

As shown in Table I, the proportion of women varied from 52.7% in the 1943–1952 birth cohort to 62.1% in the 1983–1993 birth cohort, while the proportion of white-collar workers varied from 54.1% in the 1943–1952 birth cohort to 52% in the 1983–1993 birth cohort. The highest job demands and less job control are experienced by the 1983–1993 birth cohort. At the same time, the 1973–1982 and the 1983–1993 birth cohorts appear to experience the highest levels of perceived long-lasting and sustained emotional stress as well as the poorest self-rated health compared with previous birth cohorts. The total number of person-years was 98,510.

**Table I.** Summary statistics for all variables per birth cohort based on the total number of individuals (*N*=17,730).

	Birth cohort 1943–1952	Birth cohort 1953–1962	Birth cohort 1963–1972	Birth cohort 1973–1982	Birth cohort 1983–1993
	% (*n*)/mean (SD/range)	% (*n*)/mean (SD/range)	% (*n*)/mean (SD/range)	% (*n*)/mean (SD/range)	% (*n*)/mean (SD/range)
**Number of persons**	3055	6187	5087	2726	675
**Gender**					
Women	52.7 (1609)	57.0 (3526)	57.7 (2933)	57.1 (1555)	62.1 (419)
Men	47.3 (1446)	43.0 (2661)	43.3 (2154)	42.9 (1171)	37.9 (256)
**Occupational status**					
White-collar workers	54.2 (1654)	47.6 (2947)	50.7 (2577)	52.4 (1419)	52 (351)
Blue-collar workers	45.8 (1401)	52.4 (3240)	49.3 (2510)	47.5 (1297)	48 (324)
**Job demands**	2.53 (1–4)	2.58 (1–4)	2.63 (1–4)	2.61 (1–4)	2.64 (1–4)
**Job control**	3.36 (1–4)	3.31 (1–4)	3.34 (1–4)	3.31 (1–4)	3.22 (1–4)
**Perceived long-lasting stress**	1.58 (1–4)	1.66 (1–4)	1.79 (1–4)	1.84 (1–4)	1.83 (1–4)
**Sustained emotional stress**	1.57 (1–4)	1.62 (1–4)	1.72 (1–4)	1.79 (1–4)	1.79 (1–4)
**Self-rated health**	4.02 (1–5)	3.96 (1–5)	3.99 (1–5)	4.04 (1–5)	4.12 (1–5)

### Perceived long-lasting stress, sustained emotional stress and self-rated health: age and birth cohort differences

The estimated and observed trajectories from the unadjusted models are shown in Figure 1 for perceived long-lasting stress and sustained emotional stress and in Figure 2 for self-rated health. The corresponding estimates from both the unadjusted and adjusted models for the full sample are shown in Table I while the estimates from stratified analyses are shown in Table III in Appendix 1. Models with quadratic age terms were selected for perceived long-lasting stress and sustained emotional stress. Linear age models were fitted for self-rated health, and the sustained emotional stress model for men. All variance components are statistically significant, indicating individual differences over time (Table V in Appendix 1). The overall age–cohort patterns were consistent across the unadjusted and adjusted models, although the magnitude of some estimates was attenuated after adjustment (Table I). The most recent cohorts reported more perceived long-lasting stress on average, as indicated by a positive cohort effect coefficient for the whole sample and faster increase of perceived long-lasting stress (Table I; cohort effect: 0.008; 95% confidence interval (95% CI): 0.007; 0.009; age × cohort effect: 0.009; 95% CI: 0.008; 0.106). Also, perceived long-lasting stress declines with increasing age (linear age effect: –18.507; 95% CI: –21.001; –16.014). White-collar workers report a higher decrease with increasing age (linear age effect: –21.085; 95% CI: –24.249; –17.921) compared with blue-collar workers (linear age effect: –13.568; 95% CI: –17.822; –9.315). Although this trend is statistically significant, it seems to be driven by few people in the latest cohort while the trend appears to be the opposite in the older cohorts.

Regarding overall sustained emotional stress, each successive cohort has a 0.007-unit higher stress (cohort effect: 0.007; 95% CI: 0.006; 0.007) and decreases less steeply with age (age × cohort effect: 0.006; 95% CI: 0.005; 0.007). Furthermore, emotional stress declines with increasing age (linear age effect: –11.884; 95% CI: –14.182; –9.586). White-collar workers report higher decrease with increasing age (linear age effect: –14.906; 95% CI: –17.816; –11.977) compared with blue-collar workers (linear age effect: –7.766; 95% CI: –11.799; –3.731).

Although our model estimates indicate statistically significant cohort effects overall, visual inspection suggests these effects are primarily driven by differences between the youngest cohorts. For older cohorts, patterns are less consistent and in some cases reversed, which may reflect differing age-related trajectories or contextual factors affecting these groups differently. This highlights that cohort differences in health and work environment are not uniform across all ages, and caution is warranted in generalizing results beyond the younger cohorts where data overlap and effects are clearer.

Overall self-rated health worsened with increasing age in all birth cohorts (linear age effect: –8.236; 95% CI: –11.632; –4.839). More recent cohorts (i.e. cohorts born later in time) express worse self-rated health compared with older cohorts (cohort effect: –0.003; 95% CI: –0.004; –0.002) and faster declines with age than earlier cohorts (age × cohort effect: 0.004; 95% CI: 0.002; 0.006). Figure 1 illustrates that the predicted self-rated health at the same age (vertical displacement of the lines) is slightly worse (lower) for more recent cohorts. Additionally, our results do not support significant differences in self-rated health by sex or occupational status.

### Job demands, job control: age and birth cohort effects

**Figure 3.**
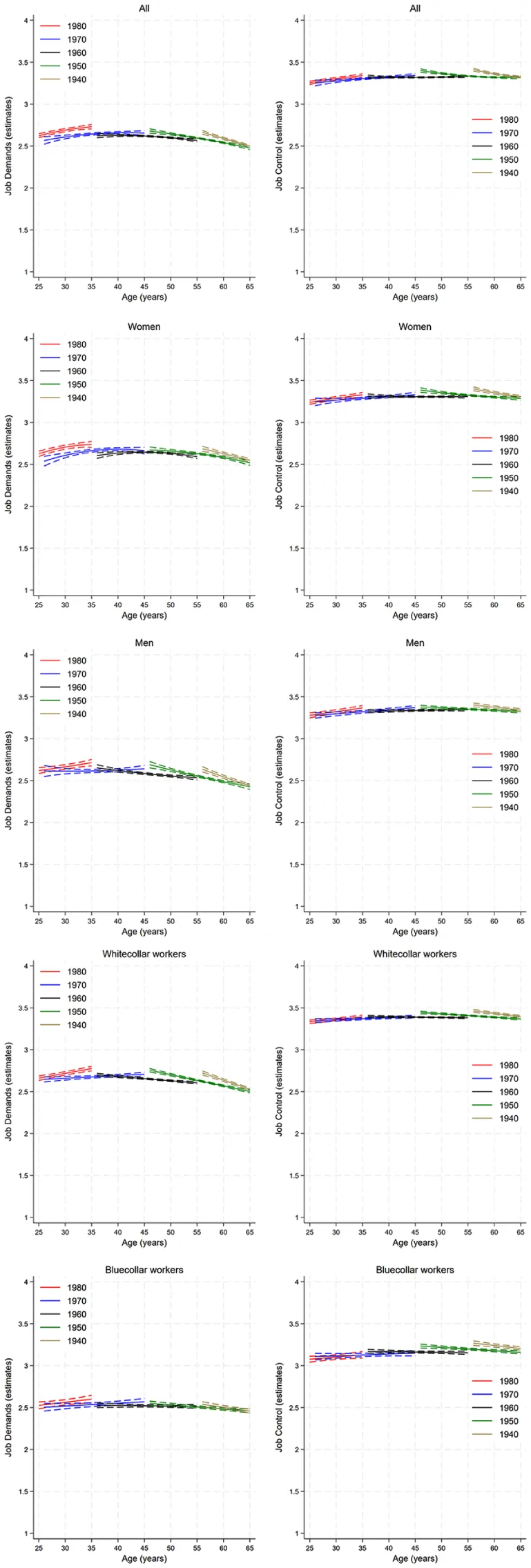
Predicted age trajectories and 95% confidence intervals for job demands and job control (range: 1–4) from the unadjusted models. The panels show trajectories for the whole sample as well as stratified by sex and occupational status. The *y*-axis represents the estimated value of the outcome variable. The cohorts are illustrated as follows: yellow line for the 1943–1952 cohort, green line for the 1953–1962 cohort, black line for the 1963–1972 cohort, blue line for the 1973–1982 cohort and red line for the 1983–1993 cohort. Data source: The Swedish Longitudinal Occupational Survey of Health, 2006–2018, total number of individuals, *N*=17,730.

**Figure 4.**
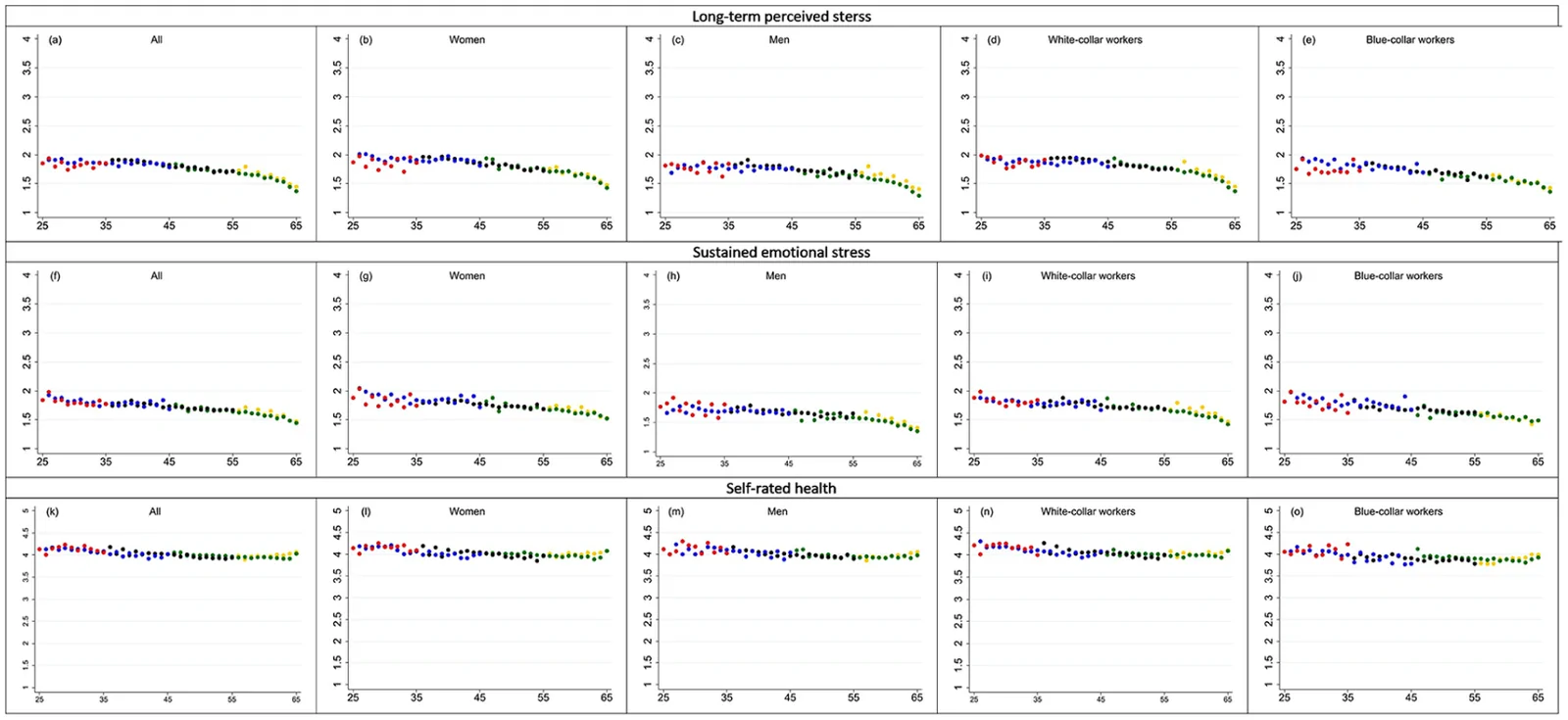
Observed age trajectories by cohort for perceived long-lasting stress, sustained emotional stress and self-rated health. The panels show trajectories for the whole sample as well as stratified by sex and occupational status: (a) long-lasting stress for all, (b) long-lasting stress for women, (c) long-lasting stress for men, (d) j long-lasting stress for white-collar workers, (e ) j long-lasting stress for blue-collar workers, (f) sustained emotional stress for all, (g) sustained emotional stress for women, (h) sustained emotional stress for men, (i) sustained emotional stress for white-collar workers, (j) sustained emotional stress for blue-collar workers, (k) self-rated health for all, (l) self-rated health for women, (m) self-rated health for men, (n) self-rated health for white-collar workers, (o) self-rated health for blue-collar workers. The *x*-axis represents age (in 10-year intervals). The *y*-axis represents the observed value of the outcome variable. The cohorts are illustrated as follows: yellow circles for the 1943–1952 cohort, green circles for the 1953–1962 cohort, black circles for the 1963–1972 cohort, blue circles for the 1973–1982 cohort and red circles for the 1983–1993 cohort. Data source: The Swedish Longitudinal Occupational Survey of Health, 2006–2018, *N*=17730.

As previously, the estimated and observed age trajectories from the unadjusted models for the full sample are shown in Figure 3. The estimated parameters from both unadjusted and adjusted models are presented in Table II. The corresponding estimates for stratified analysis are shown in Table IV in Appendix 1. Models with quadratic age effects were estimated for all outcomes except for blue-white collars and for women’s job control. The variance components are statistically significant, indicating individual differences over time (Table VI in Appendix 1). The overall age–cohort patterns were consistent across unadjusted and adjusted models, although the magnitude of some estimates was attenuated after adjustment (Table II).

**Table II.** Estimates for age and cohort effects for job demands, job control, perceived long-lasting stress, sustained emotional stress, and self-rated health from unadjusted and adjusted (for gender, occupation, education, country of birth) growth curve model estimates and 95% confidence intervals (in brackets). Swedish Longitudinal Occupational Survey of Health, 2008–2018 (*N*=17,730).

		Job demands		Job control		Perceived long-lasting stress		Sustained emotional stress		Self-rated health	
		Unadjusted	Adjusted	Unadjusted	Adjusted	Unadjusted	Adjusted	Unadjusted	Adjusted	Unadjusted	Adjusted
Intercept	γ_00_	**–3.470** (–4.826; –2.114)	**–1.469** (–4.826; –2.114)	**5.697** (4.551; 6.843)	**8.573** (7.456; 9.690)	**–13.689** (–15.154; –11.463)	**–5.234** (–7.018; –3.451)	**–11.445** (–2.484; –1.544)	**–10.860** (–12.379; –9.342)	**7.380 6.176** (4.263; 8.089) (5.501; 9.260)	
Cohort	γ_01_	**0.003** (0.002; 0.004)	**0.002** (0.001; 0.003)	**–0.001** (–0.002; –0.001)	**–0.003** (–0.003; –0.002)	**0.006** (0.007; 0.009)	**0.008** (0.002; 0.004)	**0.007** (0.006; 0.007)	**0.006** (0.006; 0.007)	**–0.003** (–0.004; –0.002)	**–0.002** (–0.003; –0.001)
Age^a^	γ_10_	**–14.648** (–17.069; –12.226)	**–13.587** ( –16.045; –11.128)	**–8.536** (–10.425; –6.648)	**–6.683** (–8.569; –4.797)	**–18.507** (–21.001; –16.014)	**–17.591** (–20.119; –15.063)	**–11.884** (–14.182; –9.586)	**–12.074** (–14.491; –9.657)	**–8.236** (–11.632; –4.839)	**–8.987** (–12.435; –5.539)
Age × cohort	γ_11_	**0.007** (0.006; 0.009)	**0.007** (0.006; 0.008)	**0.004** (0.003; 0.005)	**0.003** (0.002; 0.004)	**0.009** (0.008; 0.106)	**0.009** (0.008; 0.102)	**0.006** (0.005; 0.007)	**0.006** (0.005; 0.007)	**0.004** (0.002; 0.006)	**0.005** (0.003; 0.006)
Age-squared	γ_20_	2.482 (–1.971; 6.934)	0.406 (–4.119; 4.930)	**3.623** (0.181; 7.064)	**2.981** (0.472; 6.434)	**–14.305** (–18.945; –9.667)	**–15.903** (–20.607; –11.199)	**–10.031** (–14.266; –5.797)	**–10.868** (–15.328; –6.409)		
Age-squared × cohort	γ_21_	–0.001 (–0.003; 0.001)	–0.001 (–0.003; 0.002)	**–0.002** (–0.004; –0.01)	–**0.002** (–0.003; –0.001)	**0.008** (0.005; 0.009)	**0.008** (0.006; 0.105)	**0.005** (0.003; 0.007)	**0.006** (0.003; 0.008)		

Numbers in bold indicate significant estimates.

a Age was centred around the median age of the 10-year cohort to which the person belonged. The negative estimates are the result of the specific centring applied in the data and reflect the effects of age relative to cohort median. The actual predicted values are driven by the full combination of age and cohort effects and are constrained within the original scale for each outcome variable.

**Figure 5.**
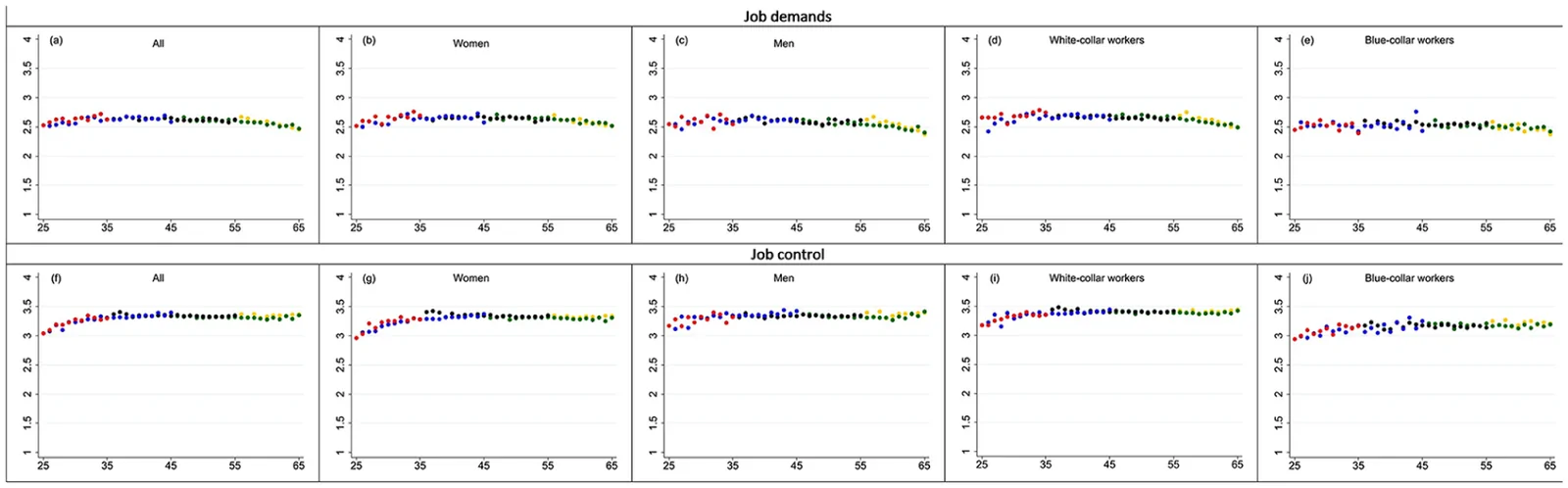
Observed age trajectories of three psychosocial work outcomes by cohort. The panels show trajectories for the whole sample as well as stratified by sex and occupational status: (a) job demands for all, (b) job demands for women (c) job demands for men, (d) job demands for white-collar workers, (e ) job demands for blue-collar workers, (f) job control for all, (g) job control for women, (h) job control for men, (i) job control for white-collar workers, (j) job control for blue-collar workers. The *x*-axis represents age (in 10-year intervals). The *y*-axis represents the observed value of the outcome variable. The cohorts are illustrated as follows: yellow circles for the 1943–1952 cohort, green circles for the 1953–1962 cohort, black circles for the 1963–1972 cohort, blue circles for the 1973–1982 cohort and red circles for the 1983–1993 cohort. Data source: The Swedish Longitudinal Occupational Survey of Health, 2006–2018, *N*=17,730.

The most recent cohorts have higher overall job demands (cohort effect: 0.003; CI: 0.002; 0.004) and less of a decrease in job demands with increasing age (age × cohort effect: 0.007; CI: 0.006; 0.009). Further, lower job demands with increasing age are observed (linear age effect: –14.648; CI: –17.069; –12.226). For instance, Figure 5(a) in Appendix 1 shows that the predicted job demands are slightly worse (higher) for those born in the cohort 1983–1993 (red dotted line) compared with those born 1973–1982 (blue dotted line). White-collar workers report a stronger decrease in job demands with increasing age (linear age effect for white-collar workers: –16.241; CI: –19.174; –13.309) compared with blue-collar workers (linear age effect for blue-collar workers: –7.969; CI: –12.327; –3.612). Men in more recent cohorts (age × cohort effect: 0.006; CI: 0.004; 0.008) and white-collar workers (age × cohort effect: 0.008; CI: 0.007; 0.009) report higher increases in job demands compared with women (age × cohort effect: 0.004; CI: 0.001; 0.003) and blue-collar workers (age × cohort effect: 0.004; CI: 0.002; 0.006).

In terms of job control, more recent cohorts report slightly less control compared with older cohorts, as indicated by the negative cohort effect estimate (cohort effect: –0.001; CI: –0.002; –0.001). The results also show that the most recent cohorts also reported slightly faster declines in job control with age than earlier cohorts (age × cohort effect: 0.004; CI: 0.003; 0.005).

Although the statistical models indicate significant cohort and age × cohort effects, visual inspection of the trajectories (e.g. Figure 3) suggests these differences are modest overall. Notably, elevated job demands and reduced job control are more apparent among the younger cohorts, while differences among older cohorts are less consistent or less pronounced and in some cases reversed. This underscores the need to interpret statistical findings in conjunction with graphical data, recognizing that cohort effects may differ by age group.

## Discussion

The present longitudinal study used APC growth-curve (unadjusted and adjusted) models to examine the effects of age and birth cohort on the trajectories of the psychosocial work environment (job demands, job control), stress experience (perceived long-lasting stress, sustained emotional stress) and self-rated health between 2008 and 2018 in a large sample that is roughly representative of the Swedish working population. To better understand the impact of demographic changes, we also examined age and cohort trajectories by gender and by occupational status (white-collar and blue-collar workers). Studies that simultaneously consider age and cohort effects are rare. To the best of our knowledge this is the first study to examine changes in job demands and job control as well as perceived long-lasting stress and sustained emotional stress by age and cohort. With regard to self-rated health, a few studies, including an earlier Swedish study, have examined changes in self-rated health by age and cohort, but the evidence is still mixed as to whether or not self-rated health deteriorates in more recent cohorts.

### Self-rated health, perceived long-lasting stress, sustained emotional stress: birth cohort and age differences

Our results support the notion that employees in the more recent cohorts have poorer self-rated health than those in earlier cohorts. This is consistent with another Swedish study showing that self-rated health has deteriorated in the youngest age groups [37], as well as an American study showing that baby boomers have lower self-rated health compared with pre-boomers [35]. Our findings are also supported by a Canadian study showing a shift from primary care to specialist care in the recent cohorts, as well as a higher likelihood of reporting multimorbidity than their predecessors [48,49]. However, our findings do not seem to be consistent with a study focusing on Swedish women, which found no significant time trends for younger women [27], and with studies outside Sweden, which found modest or no cohort effects, especially not when comparing baby-boomers with pre-boomers [34,50].

Interestingly, we found that those who are 35 years old rate their health similarly to those who are 65 years old. Extrapolating the data, this group may report worse self-rated health in the future, given the decline in self-rated health with age for all birth cohorts. Such cohort differences might arise because of advances in knowledge about health and medical care over time, for example, more recent generations have been exposed to much more health-related literature and advertising, which makes them more aware of health problems but also tends to reinforce perceptions of great personal vulnerability [35,51,52]. Another reason might be that attitudes to health might have changed over time, so that more recent cohorts might have different expectations about health and health care and might therefore rate their health status differently [35,53]. This might be related to the fact that older respondents might have lower expectations and be more tolerant of health problems than younger respondents [54] but also to the fact that individuals might rate different health domains at different ages depending on which type of health problem is considered more important at that age [55]. In the present study there is no evidence of a difference in the development of self-rated health between men and women as suggested by previous research, with women rating their self-rated health as poorer compared with men [32,37]. There was also no difference in the trends in self-rated health by occupational status. Our study is also consistent with previous studies indicating that self-rated health declines with increasing age in all cohorts [32,34,35,37,56].

In terms of perceived long-lasting stress and sustained emotional stress, our study indicates that more recent cohorts tend to report higher levels of stress compared with earlier cohorts. Also stress levels decrease with age across all cohorts. Importantly, the differences are primarily driven by younger cohorts with less consistent or even reversed patterns among older ones. This implies that the observed cohort differences in stress are not uniform across all age groups. In the present study, we cannot determine the reasons for the increasing trend in perceived and sustained emotional stress in the latest/youngest cohorts.

In contrast to previous studies reporting that young women are more stressed than men [12,57–59], we found no cohort, age or age × cohort difference in long-perceived stress and sustained emotional stress between men and women. Although there was a tendency for women in more recent cohorts to report more perceived long-lasting stress than men, the difference was not statistically significant, which is consistent with a previous study that found no difference in stress between male and female workers [60]. Our results only suggest age differences in perceived long-lasting stress and sustained emotional stress between white-collar and blue-collar workers. Previous research has produced mixed results. One study suggested that blue-collar workers had a higher risk of high perceived stress than white-collar workers [61]. The same study also found that white-collar workers who spent more than three hours per day sedentary had a greater risk of high levels of perceived stress [61]. On the other hand, another study found no difference in stress between blue-collar and white-collar workers [60]. Further, another study found no difference in emotional strain between white-collar and blue-collar workers but higher mental strain at work for blue collar workers [62]. The difference in results between our study and previous studies may be due to the assessment of stress, with the scale used here capturing cognitive stress rather than job strain, or physical stress. However, the discrepancy in the results highlights the need to study different types of stress as well as to account for cohort effects.

### Job demands and job control: birth cohort and age differences

Regarding job demands and control, the present study suggests that those in the most recent cohorts experience more job demands and less job control compared with earlier cohorts. This is in line with previous studies suggesting that job demands and resources in Sweden deteriorate or show no improvement over time [9–11]. Visual inspection of the data, however, suggests that the observed cohort effects are largely driven by comparisons among adjacent, younger cohorts, with less clear or inconsistent patterns among older groups.

In addition, our results suggest that those born in the 1960s and 1970s seem to have had a more favourable development than both older and younger generations, while the more recent and earlier cohorts seem to experience a more stressful psychosocial work environment, although the effect sizes are relatively small. Given that this value is the effect per each successive 10-year cohort, an estimate of 0.003 for job demands, for those born in 1950 and 1990, would result in a difference of 4*0.003=0.012 or 1.2 percentage points. Some previous research has suggested that younger generations are more attentive or have more negative stereotypes than older generations [63,64]. However, these cohort differences need to be replicated in other studies both in Sweden and in other countries.

Our results also support intercohort differences in the ageing trajectories of job demands by gender and occupational status. The evidence from previous research is mixed, with some studies suggesting increasing job demands for men over time, while others suggest increasing job demands for both men and women, as well as differences by industry type [13,14]. Finally, our study is consistent with some previous studies suggesting that job demands decline with age, particularly for white-collar workers [12,65]. One explanation for the decrease in job demands with age might be that older workers have more experience in coping with job demands. A previous study showed that fatigue decreases with age, which might also contribute to the decrease in job demands [12]. Another reason might be that career motives become less salient with age and therefore employees perceive fewer psychosocial work demands. The improvement in psychosocial work demands with age implies a positive situation for individuals who perceive high job demands, but might also have implications for discussions about retirement age.

In summary, our results provide some support for the intercohort change hypothesis that there are intercohort differences in the ageing trajectories of job demands, job control, stress and self-rated health across birth cohorts. While more recent cohorts report comparable or only slightly higher levels of job demands, they seem to experience more stress in response to these demands compared with earlier cohorts. However, these cohort differences seem to be primarily driven by patterns among younger cohorts with less consistent or minimal differences observed in older groups. It is likely that the work environment explains only a small part of these differences observed between more recent and earlier cohorts, as other factors might contribute to the poorer stress and subjective health perceptions among recent cohorts. Given the limited observation period and the stronger patterns found among adjacent younger cohorts, generalizations should be made with caution. Future research should further explore these cohort differences, with longer follow-up periods and additional contextual variables in order to better understand the development of health over time. Such findings might have practical implications in terms of sickness absence and health care demands. In addition, the diverse experiences across different occupational groups should be considered in future studies to provide a more comprehensive understanding of these trends.

### Strengths and limitations

The main strength of this study is that we used data from a large data sample that is approximately representative of the Swedish working population. The wide range of ages at baseline within each birth cohort allowed us to examine age and cohort effects simultaneously, using hierarchical APC-growth curve models. To the best of our knowledge, this is the first study to examine the development of the psychosocial work environment when disentangling age and cohort effects and their effects on perceived long-lasting stress and self-rated health in Sweden. In addition, we used two different measures of generic stress, derived from statistical factor analysis, namely perceived long-lasting stress and sustained emotional stress, which allows us to examine several dimensions of stress that might arise as an arousal response to work demands [66]. Finally, in this study we explored possible differential effects in the development of psychosocial work environment factors, stress and self-rated health by gender and occupational status (white-collar versus blue-collar workers), which allows us to reduce compositional effects.

However, there are some limitations to our study. One limitation is that our data did not allow us to directly account for period effects, that is, effects resulting from specific events occurring at a particular time or during a particular period. However, the statistical method used implicitly accounts for period effects, in the form of an age-by-cohort interaction [4]. Moreover, although period effects can affect people of all ages and birth cohorts, we are more interested in secular trends, which are more likely to be seen in cohort effects than in period effects. Another potential limitation might be related to the use of age-squared models, as polynomial curves are known to generate patterns that are not supported by the data, such as uplifts at each end of the time axis [67]. To mitigate this risk, we replicated the estimated trends using linear age models, which yielded consistent results. Another limitation when it comes to the study of self-rated health might be that we did not explicitly examine different health statuses. For instance, one could expect that individuals who are already severely ill might not have a significant change at the level of stress. Future work should address this issue in order to better understand which groups of employees are mostly affected. Another limitation of the study might be that the perceived stress scale used in this study measures general stress levels and therefore does not explicitly capture work-specific stress experiences. Future research should examine the contribution of both work and non-work stressors to better understand the factors driving cohort differences in perceived stress.

It is also important to note that while some cohort differences in our models were statistically significant, these differences were mainly evident in comparisons between adjacent younger cohorts. Visualizations of the predicted trajectories show that differences between older cohorts were smaller and less consistent, sometimes even reversed. This suggests that the observed cohort effects are most robust among younger birth cohorts, and caution is warranted when generalizing findings across all age groups. Studies with longer follow-up periods should replicate these findings to confirm the differences between cohorts. Finally, as SLOSH is a general population cohort it represents mainly individuals with low-level stress. Future work could examine specific subgroups of employees that are more susceptible to stress.

## Conclusion

The present study provides descriptive evidence supporting the intercohort change hypothesis, that is, that growth trajectories of job demands, job control, stress and self-rated health differ across birth cohorts. However, except for self-rated health, these differences appear to be primarily driven by comparisons between adjacent younger cohorts, whereas patterns among older cohorts were less consistent or even reversed. Across most cohorts, psychosocial job demands, stress and self-rated health improved with age. This trend might reflect factors related to ageing itself, such as accumulated work experience, as well as a positive bias effect with increasing age. With regard to cohort differences, it is possible that the more recent cohorts are exposed to more stressors, not only in the work environment but also in private and social life. To better understand these findings, further studies with longer follow-up periods are needed in order to replicate these findings both in Sweden and in other countries.

## Appendix 1. Model Estimates: Age, Cohort, Random Effects, and Goodness-of-Fit

**Table III.** Age and cohort effects on perceived long-lasting stress, sustained emotional stress and self-rated health: results from growth curve model estimates and 95% confidence intervals (in brackets) for the whole sample, women, men, white-collar workers and blue-collar workers. Swedish Longitudinal Occupational Survey of Health, 2008–2018 (*N*=17,730).

	Parameters	Women	Men	White-collar	Blue-collar
	**Perceived long-lasting stress^b^**				
Intercept	γ_00_	**–13.437** (–15.412; –2.315)	**–13.354** (–15.506; –11.203)	**–12.678** (–14.442; –10.913)	**–13.451** (–15.926; –10.976)
Cohort	γ_01_	**0.008** (0.007; 0.009)	**0.008** (0.006; 0.009)	**0.007** (0.006; 0.008)	**0.008** (0.006; 0.009)
Age^a^	γ_10_	**–18.027** (–21.384; –14.669)	**–19.167** (–22.861; –15.472)	**–21.085** (–24.249; –17.921)	**–13.568** (–17.822; –9.315)
Age × cohort	γ_11_	**0.009** (0.007; 0.108)	**0.009** (0.008; 0.011)	**0.107** (0.009; 0.123)	**0.007** (0.005; 0.009)
Age-squared	γ_20_	**–15.366** (–21.658; –9.073)	**–13.054** (–19.859; –6.249)	**–14.743** (–20.688; –8.894)	**–16.772** (–24.650; –8.894)
Age-squared × cohort	γ_21_	**0.008** (0.005; 0.011)	**0.007** (0.003; 0.010)	**0.002** (0.004; 0.105)	**0.009** (0.004; 0.126)
	**Sustained emotional stress^c^**				
Intercept	γ_00_	**–10.078** (–12.094; –8.061)	–**12.923** (–14.994; –10.852)	**–9.716** (–11.507; –7.926)	**–14.532** (–17.045; –12.018)
Cohort	γ_01_	**0.006** (0.005; 0.007)	**0.007** (0.006; 0.008)	**0.006** (0.005; 0.007)	**0.008** (0.007; 0.009)
Age^a^	γ_10_	**–13.233** (–16.367; –10.098)	**–9.639** (–12.916; –6.363)	**–14.906** (–17.816; –11.997)	**–7.766** (–11.799; –3.731)
Age × cohort	γ_11_	**0.007** (0.005; 0.008)	**0.005** (0.003; 0.007)	**0.008** (0.006; 0.009)	**0.004** (0.002; 0.006)
Age-squared	γ_20_	**–14.102** (–19.942; –8.261)		**–10.093** (–15.476; –4.712)	**–8.288** (–15.688; –0.888)
Age-squared × cohort	γ_21_	**0.007** (0.004; 0.102)		**0.005** (0.002; 0.008)	**0.004** (0.001; 0.008)
	**Self-rated health^d^**				
Intercept	γ_00_	**6.237** (3.824; 8.830)	**8.643** (5.797; 11.490)	**6.991** (4.759; 9.223)	**7.132** (3.905; 10.360)
Cohort	γ_01_	**–0.002** (–0.004; –0.001)	**–0.003** (–0.005; –0.002)	**–0.003** (–0.004; –0.001)	**–0.003** (–0.004; 0.001)
Age^a^	γ_10_	**–8.796** (–13.317; –4.276)	**–7.264** (–12.403; –2.125)	**–9.824** (–14.038; –5.609)	**–6.349** (–12.588; –0.189)
Age × cohort	γ_11_	**0.005** (0.002; 0.007)	**0.004** (0.001; 0.006)	**0.005** (0.003; 0.007)	**0.003** (0.002; 0.006)

a Age was centred around the median age of the 10-year cohort to which the person belonged. The negative estimates are the result of the specific centring applied in the data and reflect the effects of age relative to cohort median. The actual predicted values are driven by the full combination of age and cohort effects and are constrained within the original scale for each outcome variable.

b Quadratic age models were run for all, women and men; linear age models were run for white-collar and blue-collar workers.

c Quadratic age models were run for all and men; linear age models were run for women, white-collar and blue-collar workers.

d Log-linear age models were run for all outcomes.

Bold numbers indicate statistically significant estimates at the 5% level. The 95% confidence intervals are presented in brackets.

**Table IV.** Estimates^d^ of age and cohort effects for job demands, job control: results from the unadjusted growth curve model for women, men, white-collar workers and blue-collar workers. Swedish Longitudinal Occupational Survey of Health, 2008–2018 (*N*=17,730).

	Parameters	Women	Men	White-collar	Blue-collar
	**High job demands^b^**				
Intercept	γ_00_	**–1.929** (–3.478; –3.798)	**–5.234** (–7.018; –3.451)	**–2.878** (–4.395; –1.362)	–0.927 (–3.156; 1.302)
Cohort	γ_01_	**0.001** (0.001; 0.002)	**0.003** (0.002; 0.004)	**0.003** (0.002; 0.006)	**0.002** (0.001; 0.002)
Age^a^	γ_10_	**–8.090** (–11.100; –5.081)	**–11.86** (–15.378; –8.342)	**–16.241** (–19.174; –13.309)	**–7.969** (–12.327; –3.612)
Age × cohort	γ_11_	**0.004** (0.001; 0.003)	**0.006** (0.004; 0.008)	**0.008** (0.007; 0.009)	**0.004** (0.002; 0.006)
Age-squared	γ_20_	2.761 (–2.923; 8.446)	1.884 (–4.737; 8.506)		
Age-squared × cohort	γ_21_	–0.001 (–0.004; 0.001)	–0.001 (–0.004; 0.002)		
	**High job control^c^**				
Intercept	γ_00_	**6.134** (4.689; 7.578)	**5.141** (3.382; 6.899)	**6.472** (5.250; 7.693)	**9.484** (7.517; 11.455)
Cohort	γ_01_	**–0.001** (–0.002; –0.001)	**–0.001** (–0.002; –0.001)	**–0.002** (–0.002; –0.001)	**–0.003** (–0.004; –0.002)
Age^a^	γ_10_	**–9.357** (–11.802; –6.911)	**–7.568** (–10.508;–4.629)	**–6.476** (–8.635; –4.317)	**–6.078** (–9.686; –2.469)
Age × cohort	γ_11_	**0.005** (0.003; 0.006)	**0.004** (0.002; 0.006)	**0.003** (0.002; 0.004)	**0.003** (0.001; 0.005)
Age-squared	γ_20_	**6.161** (0.827; 11.495)			
Age-squared × cohort	γ_21_	–0.003 (–0.001; 0.006)			

a Age was centred around the median age of the 10-year cohort to which the person belonged. The negative estimates are the result of the specific centring applied in the data and reflect the effects of age relative to cohort median. The actual predicted values are driven by the full combination of age and cohort effects and are constrained within the original scale for each outcome variable.

b Quadratic age models were run for all, women and men; linear age models were run for white-collar and blue-collar workers.

c Quadratic age models were run for all and men; linear age models were run for women, white-collar and blue-collar workers.

d Log-linear age models were run for all outcomes.

Bold numbers indicate statistically significant estimates at the 5% level. The 95% confidence intervals are presented in brackets.

**Table V.** Random effects variance components (given as estimates and 95% CI in parentheses). and measure of goodness-of-fit for perceived long-lasting stress, sustained emotional stress and self-rated health, estimated using mixed effects models with random intercepts: a) for the whole sample, b) stratified by gender and c) stratified by occupational status (white-collar versus blue-collar workers). Swedish Longitudinal Occupational Survey of Health, 2006–2018 (*N*=17,730).

	All	Women	Men	White-collar	Blue-collar
**Perceived long-lasting stress**					
Random effects-variance components					
Level 1: within-person	**0.389** (0.386; 0.392)	**0.406** (0.402; 0.409)	**0.365** (0.360; 0.369)	**0.207** (0.205; 0.208)	**0.354** (0.349; 0.359)
Level 2: in intercept	**0.438** (0.432; 0.444)	**0.444** (0.436; 0.452)	**0.424** (0.415; 0.433)	**0.124** (0.121; 0.127)	**0.446** (0.436; 0.456)
Level 2: in growth rate	**0**.**273** (0.242; 0.312)	**0**.**275** (0.070; 0.118)	**0.268** (0.239; 0.299)	**0.098** (0.079; 0.122)	**0.256** (0.222; 0.295)
Goodness-of-fit					
BIC	88,244.13	53,485.83	34,405.63	–2163.364	26,013.7
**Sustained emotional stress**					
Random effects-variance components					
Level 1: within-person	**0.351** (0.348; 0.353)	**0.368** (0.365; 0.372)	**0.324** (0.320; 0.328)	**0.359** (0.356; 0.362)	**0.327** (0.323; 0.332)
Level 2: in intercept	**0.467** (0.461; 0.473)	**0.475** (0.467; 0.483)	**0.447** (0.438; 0.456)	**0.466** (0.458; 0.473)	**0.471** (0.461; 0.481)
Level 2: in growth rate	**0.250** (0.232; 0.269)	**0.260** (0.231; 0.294)	**0.224** (0.197; 0.254)	**0.242** (0.212; 0.275)	**0.242** (0.210; 0.278)
Goodness-of-fit					
BIC	80,460.6	49,471.91	30,424.35	54,899.75	24,430.92
**Self-rated health**					
Level 1: within-person	**0.528** (0.524; 0.532)	**0.546** (0.540; 0.551)	**0.502** (0.496; 0.508)	**0.528** (0.523; 0.533)	**0.523** (0.516; 0.531)
Level 2: in intercept	**0.594** **(0.582; 0.604)**	**0.595** (0.585; 0.606)	**0.593** (0.581; 0.606)	**0.586** (0.576; 0.596)	**0.612** (0.597; 0.626)
Level 2: in growth rate	**0.404** (0.378; 0.431)	**0.392** (0.357; 0.430)	**0.420** (0.384; 0.459)	**0.412** (0.381; 0.446)	**0.374** (0.325; 0.431)
Goodness-of-fit					
BIC	12,554.9	77,416.14	51,085.28	82,917.29	40,156.88

BIC: Bayesian Information Criterion.

Bold highlights significant variance components (*p* < 0.05).

**Table VI.** Random effects variance components (given as estimates and 95% CI in parentheses); and measure of goodness-of-fit for job demands and lack of job control, estimated using mixed effects models with random intercepts: a) for the whole sample, b) stratified by gender and c) stratified by occupational status (white-collar versus blue-collar workers). Swedish Longitudinal Occupational Survey of Health, 2006–2018 (*N*=17,730).

	All	Women	Men	White-collar	Blue-collar
**Job demands**					
Random effects-variance components					
Level 1: within-person	**0.381** (0.379; 0.384)	**0.380** (0.377; 0.381)	**0.382** (0.378; 0.387)	**0.362** (0.358; 0.365)	**0.349** (0.344; 0.354)
Level 2: in intercept	**0.310** (0.305; 0.315)	**0.311** (0.305; 0.318)	**0.304** (0.297; 0.312)	**0.394** (0.388; 0.401)	**0.419** (0.409; 0.429)
Level 2: in growth rate	**0.226** (0.201; 0.254)	**0.226** (0.201; 0.254)	**0.201** (0.169; 0.238)	**0.296** (0.274; 0.318)	**0.314** (0.284; 0.347)
Goodness-of-fit					
BIC	76,282.85	44,166.32	32,075.1	52,392.72	25,119.47
**Job control**					
Random effects-variance components					
Level 1: within-person	**0.276** (0.274; 0.278)	**0.276** (0.274; 0.279)	**0.276** (0.273; 0.279)	**0.259** (0.257; 0.262)	**0.295** (0.291; 0.299)
Level 2: in intercept	**0.335** (0.351; 0.359)	**0.354** (0.347; 0.379)	**0.356** (0.349; 0.363)	**0.330** **(**0325; 0.356)	**0.378** (0.369; 0.387)
Level 2: in growth rate	**0.239** (0.227; 0.253)	**0.238** (0.221; 0.256)	**0.240** (0.221; 0.262)	**0.219** (0.203; 0.235)	**0.223** (0.196; 0.254)
Goodness-of-fit					
BIC	51,499.23	29,618.01	21,946.44	29,364.65	19,179.64

BIC: Bayesian Information Criterion.

Bold highlights significant variance components (*p* < 0.05).

## Appendix 2: Model equations and Interpretation of the Parameters

The estimated model for our data is therefore specified by the following equations.

Level 1 repeated observation model:

$$Y_{\mathrm{ti}}=\beta_{0i}+\beta_{1i}{\mathrm{Age}}_{\mathrm{ti}}+\beta_{2i}{{\mathrm{Age}}_{\mathrm{ti}}}^{2}+e_{\mathrm{ti}}$$

Level 2 model:

For the intercept:

$$\beta_{0i}=\gamma_{00}+\gamma_{01}{\mathrm{Cohort}}_{\mathrm{ti}}+\sum \gamma_{0q}Z_{qi}+w_{0i}$$

For the linear growth rate:

$$\beta_{1i}=\gamma_{10}+\gamma_{11}{\mathrm{Cohort}}_{\mathrm{ti}}+w_{1i}$$

For the quadratic growth rate:

$$\beta_{2i}=\gamma_{20}+\gamma_{21}{\mathrm{Cohort}}_{\mathrm{ti}}+w_{2i}$$

The estimated parameters can be interpreted as follows: γ_00_ is the expected value of the outcome variables at median age for a cohort born before the median cohort; γ_01_ indicates the mean difference between consecutive 10-year; cohorts; γ_10_ and γ_20_ are the expected linear and quadratic rates of change per year; in the outcome variable in the median cohort, γ_11_ and γ_21_ are the age by cohort interaction effects and indicate mean differences in the rates of change per year between cohorts or intercohort variations in the age effects. In terms of the individual growth parameters β_0i_ and β_1i_ they reflect the expected value of the outcome at the median cohort age and the expected linear rate of increase per year of age. Finally, w_0i_, w_1i_ and w_2i_ are the residual random effects after controlling for cohort differences of person i. A more analytical description of the specification of the model can found in Yang and Land [4].
